# The role of calcium homeostasis remodeling in inherited cardiac arrhythmia syndromes

**DOI:** 10.1007/s00424-020-02505-y

**Published:** 2021-01-06

**Authors:** Shanna Hamilton, Roland Veress, Andriy Belevych, Dmitry Terentyev

**Affiliations:** Department of Physiology and Cell Biology, Davis Heart and Lung Research Institute, Wexner Medical Center, The Ohio State University, Columbus, OH USA

**Keywords:** Calcium-dependent arrhythmia, Calcium homeostasis remodeling, Heart failure, Catecholaminergic polymorphic ventricular tachycardia, Long QT syndrome

## Abstract

Sudden cardiac death due to malignant ventricular arrhythmias remains the major cause of mortality in the postindustrial world. Defective intracellular Ca^2+^ homeostasis has been well established as a key contributing factor to the enhanced propensity for arrhythmia in acquired cardiac disease, such as heart failure or diabetic cardiomyopathy. More recent advances provide a strong basis to the emerging view that hereditary cardiac arrhythmia syndromes are accompanied by maladaptive remodeling of Ca^2+^ homeostasis which substantially increases arrhythmic risk. This brief review will focus on functional changes in elements of Ca^2+^ handling machinery in cardiomyocytes that occur secondary to genetic mutations associated with catecholaminergic polymorphic ventricular tachycardia, and long QT syndrome.

## Introduction

Cardiac contractility relies on the coordinated actions of intracellular Ca^2+^ cycling machinery in cardiomyocytes, including the sarcoplasmic reticulum (SR) Ca^2+^ release channel ryanodine receptor (RyR2), SR Ca^2+^ ATPase (SERCa), the electrogenic plasmalemmal Na^+^/Ca^2+^ exchanger (NCX1), and the L-Type Ca^2+^ channel (LTCC) [6, 28]. The tight control of coupling between excitation and Ca^2+^-dependent contraction of the heart is essential for meeting variable metabolic demands of the body. Inherited mutations in ion channels, auxiliary or structural proteins that alter cardiac cell electrophysiology or cardiac conduction, manifesting as arrhythmia syndromes, usually do not dramatically change basal cardiac contractile function [28]. This strongly suggests that adaptive remodeling of intracellular Ca^2+^ transport machinery occurs to ensure long-term survival. However, under certain conditions such as stress, functional changes in Ca^2+^ handling proteins become problematic, exacerbating arrhythmia burden.

## Bidirectional control of SR Ca^2+^ release and sarcolemmal ion fluxes

During early stages of the action potential (AP), a small amount of Ca^2+^ enters the myocyte via LTCCs and NCX1 in reverse mode. This small amount of Ca^2+^ is sufficient to activate RyR2s, resulting in a massive Ca^2+^ release from the main intracellular storage organelle, the SR. Released Ca^2+^ instantaneously feeds back on sarcolemmal ionic conductance, playing important roles in shaping AP [6]. The large increase in subsarcolemmal [Ca^2+^] during the Ca^2+^ transient, which can reach 20–40 μM at its peak [71], effectively inactivates LTCCs, reducing the depolarizing force of Ca^2+^ current (*I*_Ca_). At the same time, activation of electrogenic NCX1 in forward mode, which injects 3 Na^+^ for each 1 Ca^2+^ removed from the cell, contributes to depolarization and prolongs AP duration (APD). In addition, [Ca^2+^]_*i*_ can shape AP via enhancement of Ca^2+^-dependent K^+^ and Cl^−^ channels promoting repolarization and shortening APD [39, 40]. Therefore, depending on the specific composition of ionic fluxes, SR Ca^2+^ release can either prolong or shorten APD. This is especially well illustrated during APD alternans; beat to beat alterations in APD [27]. Concordant alternans exhibit long APD when SR Ca^2+^ release is large and short when Ca^2+^ release in small. During discordant alternans, this relationship is reversed. Increase in depolarizing *I*_Ca_ and *I*_NCX_ and decrease in repolarizing currents along with untimely RyR2-mediated Ca^2+^ release cause arrhythmogenic disturbances in membrane potential called delayed or early afterdepolarizations (DADs and EADs, respectively) that underlie triggered activity at the whole heart level [64].

Pharmacological interventions to rapidly change activity of sarcolemmal ion channels and transporters are known to elicit profound effects on SR Ca^2+^ release [6]. Pharmacological inhibition of repolarizing K^+^ channels to prolong APD permits larger [Ca^2+^] influx via LTCCs, resulting in much larger Ca^2+^ release. Enhancement of Na^+^ conductance increases depolarization and induces rapid accumulation of Na^+^ in the cell, and consequently drives Ca^2+^ “overload” via inhibition of forward mode NCX1. This can result in increased amplitude of Ca^2+^ release during systole and generation of spontaneous Ca^2+^ waves during diastole. However, genetic mutations modifying the same ion fluxes often produce minimal changes in net intracellular Ca^2+^ cycling under basal conditions both in human patients and animal models. Furthermore, even mutations in components of SR Ca^2+^ release machinery are relatively well tolerated and manifestation in the form of deadly arrhythmias is a rare, primarily occurring under stress [15, 41, 69]. Therefore, constant change in electrical or mechanical properties, either acquired or inherited, or even change in activity of a single member of Ca^2+^ handling machinery must cause secondary adaptive changes that allow preservation of a primary heart function, i.e., contractility for as long as possible.

## Balance of cellular Ca^2+^ fluxes

At steady state, the amount of Ca^2+^ entering the cell via LTCCs during each beat must be equal to Ca^2+^ extruded by NCX1 [28]. Similarly, the amount of Ca^2+^ released from the SR by RyR2s must be matched by SERCa-mediated sequestration. Given the key function of rhythmic Ca^2+^ cycling in cardiomyocytes, there are several self-regulation mechanisms to maintain steady state. The most powerful mechanism is based on the ability of RyR2 to sense Ca^2+^ not only on the cytosolic side but also in the SR lumen as well. A decrease in luminal [Ca^2+^] during the Ca^2+^ transient directly or indirectly forces the cessation of RyR2 cluster activity, eliciting the termination of SR Ca^2+^ release [30, 73, 75]. Increased RyR2s activity leads to diminished SR Ca^2+^ content given the loss of Ca^2+^ during diastole, named SR Ca^2+^ leak [28]. However, this has a limited impact on the amplitude of systolic Ca^2+^ release because more active RyR2s remain open at substantially lower intra-SR [Ca^2+^]. As a result, RyR2-mediated SR Ca^2+^ leak must be sufficiently large to reduce Ca^2+^ transient amplitude. Notably, enhancement of RyR2 activity is the most common finding throughout the whole spectrum of acquired cardiac diseases including heart failure (HF), myocardial infarct (MI), diabetic cardiomyopathy, and age-related cardiac dysfunction [31, 32, 60, 84].

Another important self-limiting mechanism is Ca^2+^-dependent inactivation of LTCCs [6]. Increased *I*_Ca_ significantly increases myocyte loading, with Ca^2+^ consequently increasing systolic SR Ca^2+^ release and thereby accelerating LTCC inactivation. Pharmacologically-mediated reduction in NCX1 activity leads to similar effects on SR Ca^2+^ release and LTCC inactivation [36], which might explain why NCX1 inhibitors do not produce massive Ca^2+^ overload when used to attenuate EADs and DADs that underlie triggered activity [64]. Importantly, when the metabolic demand of the body increases, such as during stress, self-regulatory mechanisms are overridden to increase cardiac contractility [6]. During stress, the catecholamine-induced increase in LTCC-mediated Ca^2+^ influx and SERCa-mediated SR Ca^2+^ sequestration outpaces NCX1-mediated Ca^2+^ removal and RyR2-mediated diastolic Ca^2+^ leak, reaching a new steady state with increased systolic SR Ca^2+^ transient amplitude [28]. Failure to match the fluxes and deficiencies of self-regulatory mechanisms leads to impaired cardiac contractility and an enhanced propensity to Ca^2+^-dependent arrhythmia.

## Regulatory mechanisms of modulation of intracellular Ca^2+^ homeostasis

As HF is accompanied by profound changes in ionic currents and increased arrhythmogenesis, it is likely there is a substantial overlap of mechanisms underlying the remodeling of Ca^2+^ homeostasis in hereditary arrhythmia syndromes. Years of research studying remodeling of Ca^2+^ handling in HF and other models of acquired cardiac disease have revealed a number of fundamental mechanisms affecting function of Ca^2+^-handling complexes. Increased NCX1 activity in HF has been attributed to increased expression levels and an indirect effect of cytosolic Na^+^/Ca^2+^ overload given increased late Na^+^ current (*I*_NaL_) [22, 23, 65]. The expression levels of the α1c pore forming subunit of LTCC are decreased in human HF [16, 80]. However, baseline *I*_Ca_ amplitude is not affected because PKA-dependent phosphorylation of the channel, which enhances channel activity, is increased. This results in reduced responsiveness of LTCC to β-adrenergic stimulation in HF. Depressed SERCa activity in HF has been ascribed to decreased expression levels and reduced phosphorylation of auxiliary negative SERCa regulator, phospholamban (PLB) [14, 35, 79]. Increased localized activity of Serine/Threonine phosphatase PP1 underlies hypo-phosphorylation of PLB in HF [14]. This interferes with the relief of PLB’s inhibitory action on SERCa under basal conditions and during catecholaminergic stimulation. Likewise, changes in intracellular signaling cascades are involved in modulation of RyR2 activity in HF [60, 84]. Enhanced PKA- and CaMKII-dependent phosphorylation increase RyR2 activity [1, 52]. Increased RyR2 phosphorylation has been attributed to the increased activity of kinases and the dissociation of opposing phosphatases PP1 and PP2a from the complex [1, 5, 47, 54]. In addition, changes in redox state, metabolism, mitochondrial function, and subcellular structural remodeling are thought to affect Ca^2+^ homeostasis as well [60, 84]. Recent advances provide growing evidence that many of these mechanisms are similarly involved in Ca^2+^ handling remodeling in hereditary ventricular arrhythmia syndromes.

## Inherited cardiac arrhythmia syndromes and Ca^2+^ homeostasis remodeling

### Catecholaminergic polymorphic ventricular tachycardia

**Fig. 1:**
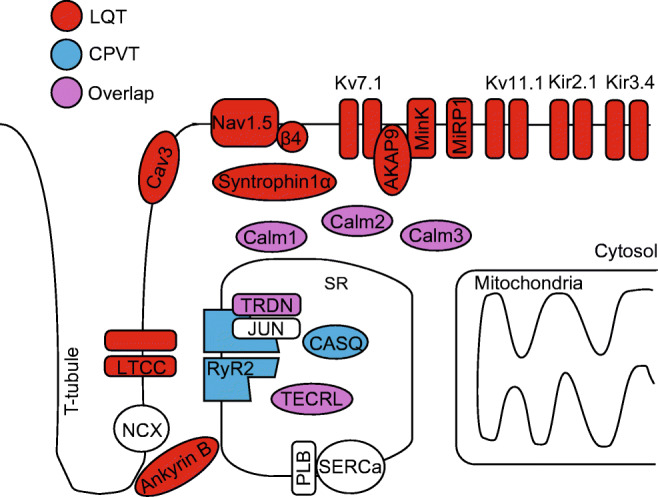
Proteins of cardiac excitation-contraction coupling associated with long QT syndrome or catecholaminergic polymorphic ventricular tachycardia, caused by pathogenic mutation. Proteins with mutations associated with long QT syndrome are colored red; proteins with mutations associated with CPVT are colored blue; proteins with mutations that can cause long QT syndrome or CPVT are colored purple. Kv7.1; *KCNQ1* gene, α-subunit of *I*_Ks_ channel, mutations underlie LQT1. Kv11.1; *KCNH2* gene, α-subunit of *I*_Kr_ channel, mutation underlies LQT2. Nav1.5; *SCN5a* gene, α-subunit of *I*_Na_ channel, mutations underlie LQT3. Ankyrin B; *ANK2* gene, functions as an adaptor protein, mutations underlie LQT4. minK; *KCNE1* gene, β-subunit of *I*_Ks_ channel, mutations underlie LQT5. MiRP1; *KCNE2* gene, β-subunit of *I*_Kr_ channel, mutations underlie LQT6. Kir2.1; *KCNJ2* gene, α-subunit of *I*_K1_ channel, mutations underlie LQT7. LTCC; *CACNA1C* gene, mutations in α-subunit of *I*_Ca,L_ channel underlie LQT8 (Timothy syndrome). Cav3; *CAV3* gene, caveolin-3 protein is a component of caveolae that co-localizes with Nav1.5, mutations underlie LQT9. β4; *SCN4B* gene, β-subunit of *I*_Na_ channel, mutation underlies LQT10. AKAP9; *AKAP9* gene, protein mediates Kv7.1 phosphorylation, mutations underlie LQT11. Syntrophin1α; *SNTA1* gene, protein regulates *I*_Na_ function, mutations underlie LQT12. Kir3.4; *KCNJ5* gene, subunit of *K*_ACh_ channel, mutations underlie LQT13. Calm1; *CALM1* gene, calmodulin serves as a Ca^2+^-binding messenger protein, mutations underlie LQT14 and CPVT4. Calm2; *CALM2* gene, mutations underlie LQT15 and phenotype overlaps with CPVT. Calm3; *CALM3* gene, mutations underlie LQT16 and CPVT6. TRDN; *TRDN* gene, triadin is an accessory protein of RyR2, mutations underlie LQT17, and phenotype overlaps with CPVT5. TECRL; *TECRL* gene, trans-2,3-enoyl-CoA reductase like protein belongs to the steroid 5-alpha reductase family, mutations underlie CPVT3 and LQT18. RyR2; *RYR2* gene, ryanodine receptor is the major sarcoplasmic reticulum Ca^2+^ release channel, mutations underlie CPVT1. CASQ; *CASQ2* gene, calsequestrin2 is an accessory protein of RyR2, mutations underlie CPVT2. JUN; *ASPH* gene, junctin is an accessory protein of RyR2, no CPVT or LQT-associated mutations reported. SERCa; *ATP2A2* gene, protein functions as the sarcoplasmic reticulum Ca^2+^-ATPase, no CPVT or LQT-associated mutations reported. PLB; *PLN* gene, phospholamban functions as an inhibitory protein of SERCa, no CPVT or LQT-associated mutations reported

Catecholaminergic polymorphic ventricular tachycardia (CPVT) is a highly malignant arrhythmogenic disorder, manifesting as polymorphic or bidirectional VT after emotional stress or exercise in patients with structurally normal hearts [15, 82]. Mutations linked to CPVT are typically associated with gain of function of RyR2 SR Ca^2+^ release complex that promotes arrhythmogenic spontaneous SR Ca^2+^ release (Fig. 1). CPVT type 1 is primarily caused by gain of function mutations in RyR2. CPVT types 2–6 have been attributed to loss-of function mutations in auxiliary proteins regulating RyR2 activity. CPVT types 2 and 5 are caused by mutations in SR luminal proteins calsequestrin (CASQ2) and triadin (TRDN) respectively [15], and characterized by loss of control of RyR2 complex activity by luminal Ca^2+^. Mutations in calmodulin (Calm) 1 and 3 (underlying CPVT types 4 and 6, respectively) and more recently Calm2, which tether to RyR2 at the cytosolic side, interfere with the complex responsiveness to activation by cytosolic Ca^2+^ [82]. CPVT type 3 has been linked to mutations in trans-2,3-enoyl-CoA reductase-like (TECRL) [24, 59], an enzyme residing primarily in the SR, but the mechanism of action is yet to be defined.

#### Ca^2+^ homeostasis and post-translational remodeling

Data accumulated over almost 20 years suggest that CPVT mutations causative of RyR2-mediated SR Ca^2+^ leak have minimal impact on Ca^2+^ transient amplitude under basal conditions. Major changes become obvious under β-adrenergic stimulation, including diminished Ca^2+^ transient amplitude and, importantly, the incidence of spontaneous diastolic Ca^2+^ waves that drive EADs and DADs [15, 41]. More direct treatment strategies targeting the RyR2 macromolecular complex that have been successfully tested using animal models include (1) pharmacological inhibition of RyR2 (dantrolene [44], flecainide [81], JTV-519 [48, 83]); (2) overexpression of WT form of accessory protein (i.e., CASQ) to reduce impact of a recessive CPVT mutation [52]; (3) gene editing-mediated disruption or siRNA-mediated suppression of a dominant CPVT mutation disease-causing allele [8, 62]; and (4) expression of exogenous plant form of regulatory protein CALM with enhanced ability to stabilize RyR2 [50].

Importantly, several indirect approaches to reduce arrhythmogenicity and improve Ca^2+^ homeostasis in CPVT models were also proven to be successful. For example, the Radwanski group reported that inhibition of late Na^+^ current is sufficient to alleviate catecholamine-induced arrhythmia in CASQ2-R33Q knock in (KI) CPVT mice [67]. Liu et al. [49] demonstrated that CaMKII inhibition with pharmacological inhibitor KN93 or inhibitory peptide AIP reduces spontaneous SR Ca^2+^ release and thereby triggered activity in the form of DADs in cardiomyocytes from RyR2-R4496C^(+/−)^ KI CPVT mice. CaMKII inhibition with KN93 completely alleviated catecholamine-induced sustained ventricular tachyarrhythmia in this model. The efficacy of CaMKII-suppression-based therapy was further validated in experiments using CPVT patient-derived iPSCs and CPVT mice with AAV-mediated overexpression of AIP [7, 25]. Moreover, experiments using CRISPR/CAS9 technology recently showed that phosphorylation at RyR2 CaMKII-specific site Serine-2814 is necessary to reveal CPVT phenotype [63]. Experiments using isolated channels from a CPVT RyR2-V2475F^(+/−)^ KI mouse model showed that phosphorylation at PKA RyR2 phosphorylation site Serine-2030 is increased in response to PKA application, while phosphorylation of CaMKII site Serine-2814 was not changed under similar conditions [51]. Taken together, these findings raise the possibility that in CPVT RyR2 complex loses association with resident phosphatases PP1 and PP2A that counter local activities of PKA and CaMKII, the phenomenon described in HF [1, 5].

#### Mitochondrial dysfunction

The information regarding CPVT-related changes in mitochondria SR-communication remains limited. Electron microscopy studies have revealed subcellular structural changes in the RyR2-A4860G^(+/−)^ mouse model of CPVT, suggesting altered tunneling and thereby communication patterns between jSR and mitochondria [45]. To our knowledge, there are no reports yet as to whether there are differences in expression levels of mitofusins, the scaffolding proteins that tether SR and mitochondria [68]; and mitochondrial Ca^2+^ handling proteins including mitochondrial Na^+^/Ca^2+^ exchanger (NCLX), and partners of mitochondrial Ca^2+^ uniporter (MCU), including Micu1, Micu2, and EMRE [29]. Our recent study [34] showed unchanged MCU expression and increased expression of MCU inhibitory paralog MCUb in CASQ2 KO CPVT mouse hearts. We demonstrated that disturbances in the RyR2 SR Ca^2+^ release complex profoundly affect mitochondrial function, causing excessive production of mitochondrial reactive oxygen species (ROS) such as superoxide and hydrogen peroxide [10, 34]. The role of less reactive of the two, hydrogen peroxide, as a second messenger is well established [72]. Given it can diffuse several microns in the cell milieu from the source [56], mitochondria-derived H_2_O_2_ can reach RyR2 clusters which are situated at a distance as close as 20 nm in ventricular myocytes [19]. Increased mito-ROS emission results in oxidation of RyR2, further increasing its activity. Importantly, mito-ROS scavenging with the mitochondrial-targeted antioxidant mito-TEMPO reduced RyR2 oxidation, restored SR Ca^2+^ content, and reduced incidence of pro-arrhythmic spontaneous Ca^2+^ waves in β-adrenergic agonist-treated cardiomyocytes from the CASQ2 knock out (KO) CPVT mouse model [34]. Earlier studies using a canine model of tachypacing-induced HF demonstrated increased RyR2 oxidation in ventricular cardiomyocytes [76]. Furthermore, mito-ROS scavenging using mito-TEMPO attenuated RyR2 oxidation and arrhythmogenic spontaneous Ca^2+^ release in a rabbit model of aging [18]. Together, these studies establish a direct link between RyR2 complex hyperactivity, RyR2 oxidation, and excessive mitochondrial-mediated ROS production, a common phenomenon in both hereditary CPVT and acquired cardiac diseases.

Of note, there is ongoing debate whether mitochondria can shape intracellular Ca^2+^ cycling serving as a Ca^2+^ buffer, in addition to being source of ROS [61]. Interestingly, pharmacological enhancement of inner mitochondrial membrane-residing Ca^2+^ uniporter (MCU) complex, or outer mitochondrial membrane residing channel VDAC, reduced spontaneous Ca^2+^ release incidence in myocytes from CPVT mice [70]. However, this beneficial effect conflicts with recent data where pharmacological facilitation of mitochondria Ca^2+^ accumulation was shown to produce mito-ROS surge, exacerbating RyR2 hyperactivity and thereby spontaneous Ca^2+^ release [33]. Furthermore, analysis of temporal parameters of spontaneous Ca^2+^ waves in this work showed that both inhibition and facilitation of mitochondrial Ca^2+^ uptake have no discernible effects on wave propagation velocity, suggesting a minimal role of mitochondria as Ca^2+^ buffer in adult ventricular myocytes [33]. Changes in Ca^2+^ wave incidence and frequencies caused by facilitation and inhibition of mito-Ca^2+^ uptake reported in this manuscript were attributable to the changes in RyR2 oxidation levels by mito-ROS. These results are in line with the view accepted by several leading groups that mitochondria Ca^2+^ buffering ability in terminally differentiated VMs is very low in comparison to contractile apparatus or SERCa [4, 9, 28, 53].

#### Subcellular structural remodeling

Typically CPVT mutations do not cause structural remodeling of the heart [15]. However, there is a growing evidence of CPVT-associated changes in ventricular myocyte subcellular organization. The first indications of such phenomena have been obtained using CASQ2 KO CPVT mouse model where Knollmann et al. [43] documented dramatic increase in SR volume, potentially a compensatory change to preserve SR Ca^2+^ buffering capacity in the absence of CASQ2, a major luminal Ca^2+^ buffer. An elegant study from this group which followed demonstrated that KO of luminal accessory protein TRDN (to mimic CPVT-linked loss-of TRDN-function mutations) causes profound changes in RyR2 complexes and subcellular structural organization, leading to almost 50% loss of contacts between T-tubules and junctional SR [17]. Loss of contacts between T-tubules and jSR is a recurrent finding in HF [12, 16, 74]. Importantly, prevention of proteasomal degradation of misfolded proteins by an inhibitor of mannosidase-I kifunensine successfully reduced CPVT occurrence in TRDN-KO mice [13]. The loss of jSR-T-tubular contacts in TRDN KO cardiomyocytes results in reduced Ca^2+^-dependent inactivation of LTCCs, enhancing Ca^2+^ influx through the plasmalemma. Interestingly, later studies revealed that CPVT linked to TRDN mutations exhibit features consistent with long QT syndrome (LQTS) as well [2], which is not surprising given LTCC inactivation impairment. The overlap with LQTS was also noticed for CPVT TECRL loss-of function mutations manifested by QTc prolongation in patients under catecholaminergic surge [24, 59].

Taken together, these works provide strong support for the concept that initial insult by CPVT mutations causes profound secondary changes in the following: (a) posttranslational control of RyR2 activity; (b) mitochondrial function; and (c) intracellular structural organization. Evidently, these secondary changes are key to revealing the arrhythmogenic phenotype in CPVT.

### LQT syndrome and Ca^2+^ release

Long QT syndrome (LQTS) is a malignant arrhythmogenic disorder, characterized by QT prolongation accompanied with ventricular tachyarrhythmias typically in the form of torsade de pointes (TdP) and polymorphic VT [3, 15, 41, 69]. Arrhythmic events in LQTS usually occur in patients during emotional stress or exercise and less frequently during sleep. Mutations in three genes are responsible for the vast majority of LQTS cases in humans, namely *KCNQ1* encoding Kv7.1 channel α-subunit (LQT1, 35% of cases), *KCNH2* encoding Kv11.1 channel α-subunit (LQT2, 30% of cases), and *SCN5A* encoding Nav1.5 Na^+^ channel α-subunit (LQT3, 10% of cases). Loss-of-function K^+^ channel mutations reduce repolarizing K^+^ currents *I*_Ks_ (LQT1) and *I*_Kr_ (LQT2) leading to AP prolongation, similarly to gain-of-function LQT3 mutations in Na^+^ channel which promote depolarization. As mentioned above, acute pharmacologically induced AP prolongation in ventricular myocytes leads to severe intracellular Ca^2+^ overload, enhancing both systolic and arrhythmogenic spontaneous SR Ca^2+^ release. Increased Ca^2+^ transient amplitude increases cardiac contraction. However, a robust increase in cardiac function is not a common observation in inherited LQTS. Available literature documents mechanical changes in human LQTS patients and large animal models consistent with diastolic dysfunction [41, 69], which implies adaptive remodeling of Ca^2+^ homeostasis occurs. Given HF is accompanied by a loss of repolarizing currents and increase in *I*_NaL_, the mechanisms underlying changes in Ca^2+^ handling may have substantial overlap with those in LQTS.

#### LQT2

Notably, *I*_Ks_ and *I*_Kr_ have minimal roles in repolarization in rodents [6]. Therefore, the studies using large animal models of LQT1 and LQT2 provide vital opportunities to delineate arrhythmia mechanisms and potential role of changes in Ca^2+^ homeostasis secondary to mutation-induced AP prolongation [3]. Transgenic rabbits overexpressing LQT2-linked mutant *KCNH2* (HERG-G628S) in the heart exhibited significant AP prolongation and high incidence of SCD (> 50% at 1 year) due to polymorphic VT, recapitulating human LQTS [11]. Experiments using isolated ventricular myocytes from LQT2 hearts revealed decrease in SR Ca^2+^ content and Ca^2+^ transient amplitude, particularly noticeable under β-adrenergic stimulation [77]. Further analysis showed unchanged *I*_Ca_ and NCX1 function, while SR SERCa-mediated Ca^2+^ uptake and RyR2-mediated SR Ca^2+^ leak were accelerated in LQT2 ventricular myocytes. Increased SERCa activity in LQT2 has been attributed to an increase in PKA PLB phosphorylation under baseline conditions [77]. Typically SERCa activity is reduced in HF; however, increased PLB phosphorylation was previously reported in a rabbit pressure-overload-induced model of HF [20]. Enhanced RyR2 activity in LQTS has been ascribed to an increase in PKA and CaMKII phosphorylation of the channel due to the loss of phosphatases PP1 and PP2a from the complex. Identical results were reported earlier in rabbit and canine HF models [1, 5].

Enhanced RyR2-mediated loss of SR Ca^2+^ during diastole facilitates NCX1-mediated Ca^2+^ removal to balance increased LTCC-mediated influx during longer AP [6]. More active SERCa plays a primary role in shortening the Ca^2+^ transient during AP plateau when membrane potentials are close to NCX1 reversal potential. Together, these events prevent a substantial increase in Ca^2+^ transient amplitude in LQT2 ventricular myocytes under basal conditions, in contrast to pharmacological *I*_Kr_ block. However, in the presence of β-adrenergic agonist isoproterenol, enhanced RyR2 activity becomes the major contributor to triggered activity in the form of arrhythmogenic EADs [67]. Stabilization of RyR2 function by pharmacological inhibition of CaMKII is sufficient to completely alleviate Ca^2+^-dependent afterdepolarizations in LQT2 ventricular myocytes [67]. Partial inhibition of NCX1 activity either directly by using pharmacological NCX inhibitor SEA400 [55] or indirectly by blocking Late *I*_Na_ with GS967 [37] also effectively eliminates EADs in this model. However, chronic use of NCX1 or *I*_NaL_ inhibitors for arrhythmia prevention in LQT2 requires extensive testing to ensure no adverse effects of such treatments.

The data whether or LQT1 or 2 induces subcellular structural remodeling is lacking. However, proteomics analysis demonstrated significant changes in expression levels and activities of enzymes involved in ATP generation via glucose utilization and fatty acids β-oxidation pathways [38], suggesting increased energy demand and increased supply in LQT1 and LQT2 transgenic rabbit hearts.

#### LQT3

LQT3 is associated with *SCN5A* gain-of-function mutations that impede inactivation of the channel, leading to increased *I*_NaL_ [3, 11, 41]. Unlike most LQTS, arrhythmia episodes in LQT3 occur during sleep or rest in the absence of increased catecholaminergic tone. A decrease in heart rhythm provokes profound lengthening of AP and increases incidence of tdP and polymorphic VTs in human patients. Pharmacological *I*_NaL_ enhancement to model LQT3 in rabbit ventricular myocytes induces intracellular Na^+^/Ca^2+^ overload, which accelerates mitochondrial ROS production [78]. Increased ROS leads to oxidation and thereby activation of CaMKII. Activated CaMKII phosphorylates RyR2 increasing its activity, which underlies an increase in pro-arrhythmic spontaneous Ca^2+^ release. In this work, both antioxidants and CaMKII inhibition restored diminished Ca^2+^ transients and reduced diastolic [Ca^2+^] and spontaneous Ca^2+^ waves, similar to the effects in mouse ventricular myocytes with pressure-overload induced HF [78]. Experiments using LQT3 mutation mouse models suggest that increased *I*_NaL_ increases SR Ca^2+^ load and this increase promotes arrhythmogenic spontaneous waves [46]. Interestingly, in mice with LQT3 evoked by deletion residues 1510–1512 (ΔQKP) in the *Scn5a* gene, SERCa activity was depressed due to increased PLB expression and its reduced phosphorylation [58]. Furthermore, NCX1 expression and activity were unaltered. This is an interesting finding because Na^+^ overload is expected to impede forward mode NCX1. The simplest explanation of these phenomena is that *I*_NaL_ enhancement is insufficient to significantly alter intracellular [Na^+^] despite the profound effect on APD. If this is the case, prolonged LTCC-mediated Ca^2+^ influx during the long AP is sufficient to increase SR Ca^2+^ content in LQT3. Indeed, mouse models of LQT8 (Timothy Syndrome) linked to LTCC gain-of-function mutations in *CACNA1C* also show increase in SR Ca^2+^ content and increased frequency of spontaneous Ca^2+^ waves in ventricular myocytes [26]. Remarkably, in the *Scn5a* ΔQKP LQT3 model, RyR2 phosphorylation remained unchanged and no evidence of enhanced activity of the channel was presented despite an increase in spontaneous Ca^2+^ waves [58], which is not the case in HF. However, LQT3 Ca^2+^ transients exhibited longer time-to-peak, suggesting subcellular dyadic structural remodeling: a hallmark of HF.

Notably, to our knowledge, a large animal model of hereditary LQT3 is yet to be created. Given substantial differences in Ca^2+^ cycling patterns between mice and larger animals, mechanisms of secondary remodeling uncovered in mice may differ greatly than those in humans. In small rodents, an increase in stimulation frequency decreases Ca^2+^ transient amplitude, e.g., a negative staircase. In rabbits or humans, increased stimulation frequency increases Ca^2+^ transients [6]. Accordingly, the SR loses Ca^2+^ at slower rates due to higher NCX1 activity and lower SERCa activity in large animals and humans vs mice. Therefore, given that arrhythmia episodes in human LQT3 patients are prevalent during slower heart rates and assuming that spontaneous Ca^2+^ release is a key element of trigger [58], there is a good chance that SERCa activity is increased, in stark contrast to mice. Indeed, Xiao Yan Qi et al. [66] showed enhanced SERCa activity and PLB phosphorylation due to enhanced activity of CaMKII in rabbit hearts with slowed heart rate induced by AV block 2 weeks after the procedure. At the cellular level, bradycardia was accompanied by AP prolongation resulting in enhanced LTCC-mediated Ca^2+^ influx, increased SR Ca^2+^ load, increased Ca^2+^ transient amplitude, increased contraction, and importantly, arrhythmogenic EADs at very slow pacing rates in the absence of β-adrenergic stimulation. At higher stimulation rates, presence of β-agonist was necessary for EADs induction.

#### Other inherited arrhythmia syndromes

The list of genes associated with LQTS is rapidly expanding. Although most of these genes encode proteins that regulate K^+^ and Na^+^ conductance, the list of mutations in genes directly involved in Ca^2+^ handling that manifest as LQTS continues to grow. *Calm1*, *Calm2*, *Calm3*, *TRDN*, and *TECRL* (LQT14–18) are recent additions to the *CACNA1C* gain-of-function mutations associated with LQT8 [2, 24, 26, 41, 58] (Fig. 1). Although much remains to be done to delineate specific mechanism underlying electrical defects triggered by these mutations, it is unequivocally obvious how tightly changes in electrical activity are coupled with changes in Ca^2+^ handling. Furthermore, the key roles of secondary to initial insult Ca^2+^ remodeling become widely recognized in other forms of hereditary arrhythmias including Arrhythmogenic Right Ventricular Hypertrophy [21] and Brugada Syndrome [57].

## Perspective

**Fig. 2:**
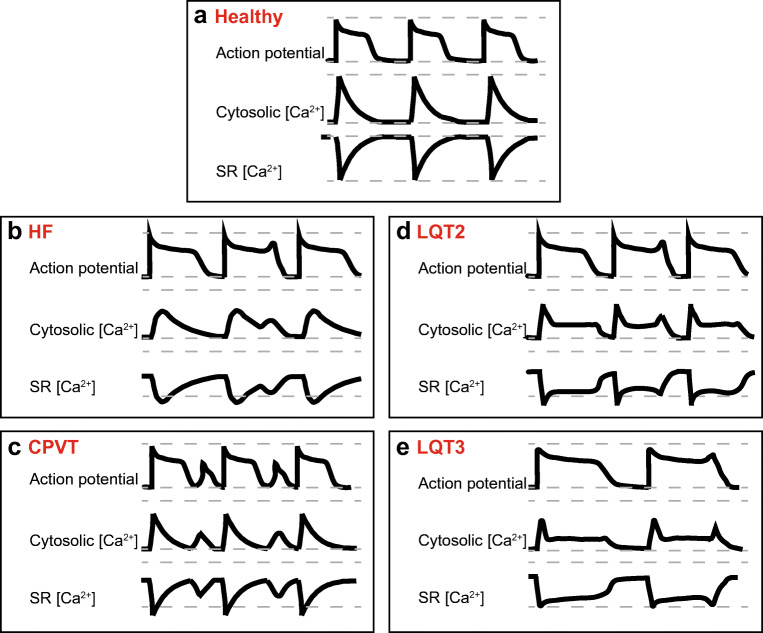
Comparison of proarrhythmic changes in action potentials and Ca^2+^ homeostasis in HF, CPVT, and LQTS 2 and 3 ventricular myocytes. **a** Schematic of action potentials, Ca^2+^ transients, and changes in intra-SR Ca^2+^ in a healthy human ventricular myocyte under β-adrenergic stimulation. Grey dashed lines indicate minimum and maximum Ca^2+^ levels reached in healthy myocytes. **b** In HF, APD is prolonged due to decrease in K^+^ currents and increase in late *I*_Na_. Ca^2+^-dependent EADs/DADs underlie arrhythmogenesis under β-adrenergic stimulation. Enhanced sensitivity of RyR2 to intra-SR [Ca^2+^] due to increased phosphorylation and oxidation of the channel leads to termination of systolic Ca^2+^ release at reduced intra-SR [Ca^2+^]. Faster RyR2-mediated SR Ca^2+^ leak and reduced refractoriness of RyR2 also contributes to the enhanced propensity for proarrhythmic spontaneous Ca^2+^ release. Enhanced NCX1 activity, depressed SERCa activity and SR Ca^2+^ leak underlie reduced intra-SR [Ca^2+^] and diminished Ca^2+^ transient amplitude. Loss of dyadic contacts between T-tubular LTCCs and jSR RyR2s impedes Ca^2+^ transient rise. **c** Under β-adrenergic stimulation, CPVT myocytes exhibit spontaneous Ca^2+^ release via defective RyR2 complexes, leading to reduced Ca^2+^ transient amplitude and reduced intra-SR [Ca^2+^]. Posttranslational remodeling, mitochondrial dysfunction, and subcellular structural remodeling contribute to the hyperactivity of RyR2 caused by CPVT-associated mutations. Proarrhythmic activity of RyR2 drives NCX1 activity, causing a depolarizing inward current and DADs. Uncoupling of LTCCs and RyR2s due to dyad remodeling may increase Ca^2+^ transient rise time and reduce LTCC Ca^2+^-dependent inactivation which can result in longer APD. **d** In LQT2, loss-of-function mutation in KCNH2 reduces outward *I*_Kr_ and prolongs APD during β-adrenergic stimulation. SR Ca^2+^ leak is accelerated due to hyperphosphorylation of RyR2. SERCa-mediated SR Ca^2+^ uptake is accelerated at baseline due to PLB phosphorylation. Enhanced activity of hyperphosphorylated RyR2s contributes to a reduction of SR [Ca^2+^], Ca^2+^ transients amplitude, and arrhythmogenic EADs under β-adrenergic stimulation. **e** In LQT3, gain-of-function mutation in SCN5A increases inward late *I*_Na_ and prolongs APD. Arrhythmogenic activity occurs at rest, in the absence of β-adrenergic stimulation. Longer APD increases LTCC-mediated Ca^2+^ influx. Na^+^/Ca^2+^ overload and increased activity of SERCa due to PLB phosphorylation underlies increase in SR Ca^2+^ content, Ca^2+^ transient amplitude, and spontaneous RyR2-mediated Ca^2+^ release thereby EADs at slow rates

Sudden cardiac death remains a major health problem in the postindustrial world. Over the last quarter of century, significant progress has been made in identification of genetic components of malignant cardiac arrhythmia and improved diagnostics. This lead to rapid development of effective therapies; however, further advancement in this area requires a significantly new level of mechanistic understanding. The body of evidence accumulated over the last decade provided strong foundation for a new paradigm-shifting concept when it became obvious that the impact of a single point mutation goes far beyond elementary modification of a certain enzyme or ion channel function. Instead, mutation can induce systemic changes affecting numerous cellular signaling cascades, energy production, protein expression and degradation, and Ca^2+^ homeostasis. This remodeling, in an attempt to provide long-term preservation of basic contractile cardiac function, ultimately exacerbates arrhythmic potential under certain conditions such as stress. Notably, since the main goal is the same, i.e., preservation of contractile function, remodeling pathways evoked by arrhythmogenic mutations in genes encoding proteins involved in Ca^2+^ transport, cell electrical activity, or structural elements that underlie cardiac conduction often converge, bearing resemblance of those in structural heart disease and each other (see Fig. 2).

Given that arrhythmia syndromes are accompanied by cell systems remodeling, there is great promise in expansion of classical reductionist approaches with rapidly developing new techniques including proteomics, transcriptomics, metabolomics, and big data analytical tools to identify druggable nodes. This is expected to facilitate development of brand new classes of antiarrhythmic agents with improved efficacy and reduced adverse effects. The understanding that remodeling secondary to initial insult caused by a specific mutation has an enormous impact on arrhythmogenesis points to a necessity for expanded genetic screening panels of patients with idiopathic arrhythmias to a new level far beyond classical suspects. Also, although valuable information is being obtained generated using patient-induced pluripotent stem cell (iPSC)–derived cardiomyocytes, this experimental platform needs further development to ensure the highest maturation degree of subcellular structure, metabolic, and signaling cascades, given their key roles in revealing arrhythmogenic phenotype [42]. Finally, the value in future development of engineered tissues and large animal models of hereditary arrhythmias to study mechanisms and test antiarrhythmic therapies cannot be overstated.
